# Identification of glucocorticoid-related genes in systemic lupus erythematosus using bioinformatics analysis and machine learning

**DOI:** 10.1371/journal.pone.0319737

**Published:** 2025-03-25

**Authors:** Yinghao Ren, Weiqiang Chen, Yuhao Lin, Zeyu Wang, Weiliang Wang

**Affiliations:** 1 Department of Dermatology, Xiamen Humanity Hospital Fujian Medical University, Xiamen, Fujian, China; 2 Department of Nephrology, Xiamen Humanity Hospital Fujian Medical University, Xiamen, Fujian, China; 3 Department of Endocrinology, Xiamen Humanity Hospital Fujian Medical University, Xiamen, Fujian, China; 4 State Key Laboratory of Holistic Integrative Management of Gastrointestinal Cancers and National Clinical Research Center for Digestive Diseases, Xijing Hospital of Digestive Diseases, Fourth Military Medical University, Xi’an, Shaanxi, China; 5 Epilepsy Center, Xiamen Humanity Hospital Fujian Medical University, Xiamen, Fujian, China; Arizona State University, UNITED STATES OF AMERICA

## Abstract

**Background:**

Systemic lupus erythematosus (SLE) is a complex autoimmune disease that has significant impacts on patients’ quality of life and poses a substantial economic burden on society.

**Objective:**

This study aimed to elucidate the molecular mechanisms underlying SLE by analyzing glucocorticoid-related genes (GRGs) expression profiles.

**Methods:**

We examined the expression profiles of GRGs in SLE and performed consensus clustering analysis to identify stable patient clusters. We also identified differentially expressed genes (DEGs) within the clusters and between SLE patients and healthy controls. We conducted Gene Set Enrichment Analysis (GSEA) and Gene Set Variation Analysis (GSVA) to investigate biological functional differences, and we also conducted CIBERSORTx to estimate the number of immune cells. Furthermore, we utilized least absolute shrinkage and selection operator (LASSO) regression and Random Forest (RF) algorithms to screen for hub genes. We then validated the expression of these hub genes and constructed nomograms for further validation. Moreover, we employed single-sample Gene Set Enrichment Analysis (ssGSEA) to analyze immune infiltration. We also constructed an RNA-binding protein (RBP)-mRNA network and conducted drug sensitivity analysis along with molecular docking studies.

**Results:**

Patients with SLE were divided into two subclusters, revealing a total of 2,681 DEGs. Among these, 1,458 genes were upregulated, while 1,223 were downregulated in cluster_1. GSVA showed significant changes in the pathways associated with cluster_1. Immune infiltration analysis revealed high levels of monocyte in all samples, with greater infiltration of various immune cells in cluster_1. A comparison of SLE patients to control subjects identified 269 DEGs, which were enriched in several pathways. Hub genes, including PTX3, DYSF and F2R, were selected through LASSO and RF methods, resulting in a well-performing diagnostic model. Drug sensitivity and docking studies suggested F2R as a potential new therapeutic target.

**Conclusion:**

PTX3, DYSF and F2R are potentially linked to SLE and are proposed as new molecular markers for its onset and progression. Additionally, monocyte infiltration plays a crucial role in advancing SLE.

## Introduction

Systemic lupus erythematosus (SLE) is a complex autoimmune disorder characterized by a wide range of clinical manifestations that can affect multiple organ systems [1]. Its etiology remains incompletely understood, but it is known to have significant implications for patient quality of life and healthcare systems worldwide [2]. The burden of SLE is not only evident in the clinical challenges it poses but also in the economic impact related to medical care and productivity losses associated with the disease [3]. Current therapeutic strategies, primarily involving glucocorticoids and immunosuppressants, show considerable variability in efficacy among individuals, underscoring the need for more tailored treatment approaches [4].

Previous research has focused extensively on the genetic and immunological underpinnings of SLE [5]. Studies have identified various biomarkers and genetic signatures associated with disease onset and progression, yet the complexity of the disease and the heterogeneity of patient responses to existing therapies highlight significant gaps in our understanding [6]. The interplay between genetic predispositions, environmental factors, and immune dysregulation remains an active area of investigation, necessitating further exploration into the molecular mechanisms driving SLE pathogenesis [7].

Among the critical areas of research is the expression of GRGs, which have been shown to play crucial roles in the disease’s pathophysiology [8]. Altered expression of these genes may not only influence disease activity but also affect responses to glucocorticoid therapy [9]. Understanding these gene expressions can provide insights into the mechanisms of action of glucocorticoids and their impact on immune responses in SLE [10].

We employed advanced bioinformatics approaches and machine learning, including consensus clustering, differential expression analysis and immune infiltration analysis, to systematically investigate the gene expression profiles in SLE patients. These methodologies enable the integration of large-scale genomic data, facilitating the identification of hub genes and pathways implicated in SLE pathogenesis [11]. The aim is to elucidate the molecular mechanism of SLE and uncover new biological markers that could enhance diagnostic and therapeutic strategies, ultimately leading to improved patient outcomes.

## Methods

### Data source

We downloaded full-genome expression profiles for SLE from the Gene Expression Omnibus (GEO) database [12]. Dataset GSE144390 included 3 peripheral blood mononuclear cell (PBMC) samples from SLE patients and 3 from healthy controls [13], while GSE50772 had 61 SLE patient PBMC samples and 20 healthy control PBMC samples [14].

We applied the ComBat method from the R package “sva” to adjust for batch effects and evaluated how well the correction worked using principal component analysis [15].

**Fig 1:**
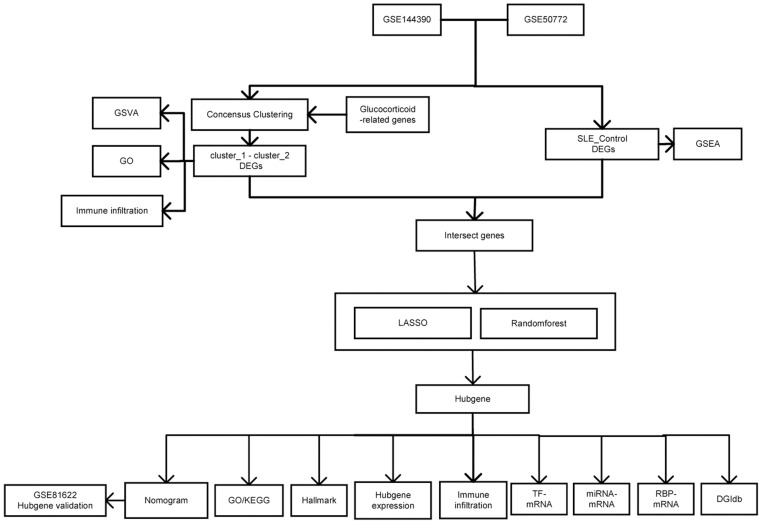
The flowchart of this study.

The external validation dataset GSE81622 included 55 samples, comprising 25 PBMC samples from SLE patients and 15 from healthy controls [16]. GRGs were downloaded from Molecular Signatures Database (MSigDB) [17] (S1 Table). The workflow of this study was illustrated in Fig 1.

### Consensus clustering analysis

We applied the gene expression matrix from the SLE dataset to identify different SLE clusters based on GRGs using the consensus clustering method in the R package “ConsensusClusterPlus” [18]. The model was repeated 1,000 times, with k set to 2, ensuring the stability of the clustering. The optimal clustering was identified using the cumulative distribution function (CDF), which showed the slowest decrease.

### Differential analysis among subclusters and SLE-related DEGs

We used the R package “limma” to identify DEGs in two comparisons: between Cluster_1 (n = 39) and Cluster_2 (n = 25), and between the control group (n = 23) and the SLE group (n = 64) [19]. We established the selection criteria as | log2Fold Change | > 1 and adjusted p < 0.05. These DEGs were used for subsequent analyses. Heatmaps were generated using the R package “pheatmap”, employing Euclidean distance and hierarchical clustering methods.

### Enrichment analysis

In this study, differential expression analysis between SLE and healthy controls was performed using the R package “limma”, calculating the fold change (FC) of gene expression in SLE relative to healthy controls [20]. GSEA was performed using the R package “clusterProfiler”, analyzing the ordered list of all genes ranked by log2FC. Each analysis included 1,000 gene set permutations. The c2.cp.kegg.v7.5.1.symbols gene set was used as the reference, stored in the MSigDB [21]. After correction, gene sets with a p-value < 0.05 (pAdjustMethod = “BH”, pvalueCutoff = 0.05) and a normalized enrichment score (NES) >  1.5 were considered significantly enriched.

To explore the biological functional differences between the control group and the SLE group, we used the “c2.cp.kegg.v7.5.1.symbols” gene set from the MSigDB as the reference. We visualized the results with the R package “pheatmap”. Additionally, 50 hallmark gene sets were downloaded from the MSigDB to serve as reference gene sets, and the ssGSEA function within the Gene Set Variation Analysis (GSVA) package was used to calculate the GSVA scores for each gene set across different samples. Differences in GSVA scores between the control group and the SLE group were analyzed using the “limma” package.

Gene Ontology (GO) analysis encompasses biological processes (BP), molecular functions (MF), and cellular components (CC) [22]. The Kyoto Encyclopedia of Genes and Genomes (KEGG) is employed to identify significant changes in metabolic pathways enriched in gene lists [23]. The R package “clusterProfiler” was applied to perform GO annotation and KEGG pathway enrichment analyses (p < 0.05) on glucocorticoid-related DEGs [24].

### Identification of hub genes

We used the “glmnet” package in R to perform LASSO regression [25]. We used a binomial distribution for LASSO classification, establishing the model by selecting the minimum error. The model performed well, although it was limited to only 10 cross-validation variables. The “RandomForest” (RF) function was employed for RF analysis. We selected the minimum error as the mtry node value and chose the stable image value as ntree. We selected the top 10 hub genes based on the Mean Decrease in Accuracy (MDA) and Mean Decrease in Gini (MDG) of feature importance. These genes were identified as significant by the RF analysis. In this study, we combined LASSO regression and RF analysis to select the most significant hub genes.

### Construction and validation of a nomogram for SLE diagnosis

We developed a nomogram for diagnosing SLE using the R package “rms”. Risk scores were computed from the expression levels of hub genes, with the total risk score being the sum of these individual scores. We evaluated the nomogram’s diagnostic value for SLE using calibration and ROC curves. We also validated the nomogram model using ROC curves from the GSE81622 dataset.

### CIBERSORTx analysis

We used CIBERSORTx to estimate the relative abundance of immune cells from gene expression datasets (https://cibersortx.stanford.edu/) [26]. The CIBERSORTx algorithm operated under batch mode for batch correction and relative mode with 1,000 permutations. Differences between subclusters were analyzed using the Wilcoxon rank-sum test.

### Immune infiltration analysis

Single Sample Gene Set Enrichment Analysis (ssGSEA) is an extension of GSEA that calculates enrichment scores for each sample and gene set [27]. We downloaded data on 28 types of immune cells from the Tumor Immune System Interaction Database (TISIDB) [28]. Relative enrichment scores for each immune cell were calculated from the gene expression profiles of each sample. The variation in levels of immune cell infiltration between the SLE and control groups was depicted using the R package “ggplot2” [29].

### Construction of the RBP-mRNA network

This study utilized the open-source platform StarBase to analyze ncRNA interactions (https://starbase.sysu.edu.cn/tutorialAPI.php#RBPTarget). We employed CLIP-seq, degradome-seq, and RNA-RNA interaction data to explore the associations between mRNA and RBP expressions. In the disease, p < 0.05, clusterNum ≥ 5, and clipExpNum ≥ 5 were defined as the cutoff criteria for identifying key mRNA-RBP pairs. We then constructed the RBP-mRNA network using Cytoscape [30].

### Drug sensitivity analysis

We utilized the Drug-Gene Interaction Database (DGIdb, http://www.dgidb.org/) to explore and collect drug-gene interaction information for specific genes [31]. DGIdb is a database that integrates a variety of publicly available and licensed drug-gene interaction data, which plays a significant role in research on drug targets and personalized medicine.

### Molecular docking

The 3D structures of the small molecules were downloaded from the PubChem database (https://pubchem.ncbi.nlm.nih.gov) [32]. Key target proteins were retrieved from the Protein Data Bank (PDB) database (http://www.rcsb.org) [33]. Only high-resolution structures were selected. The 3D structures of these proteins were downloaded and imported into PyMOL 2.5 to remove water and ligands [34]. Subsequently, docking was performed using AutodockVina software and the 3D structures of the small molecules [35]. The lowest binding energy values were recorded along with the structural files, which were then visualized using PyMOL.

### Statistical analysis

We conducted statistical analyses using R software version 4.1.2. We used the Spearman correlation test to assess relationships between two parameters. We assessed differences between two groups using the Wilcoxon test. For comparing differences among three or more groups, we applied the Kruskal-Wallis test. A two-sided p < 0.05 was considered statistically significant.

## Results

### Differential analysis among the subclusters

**Fig 2:**
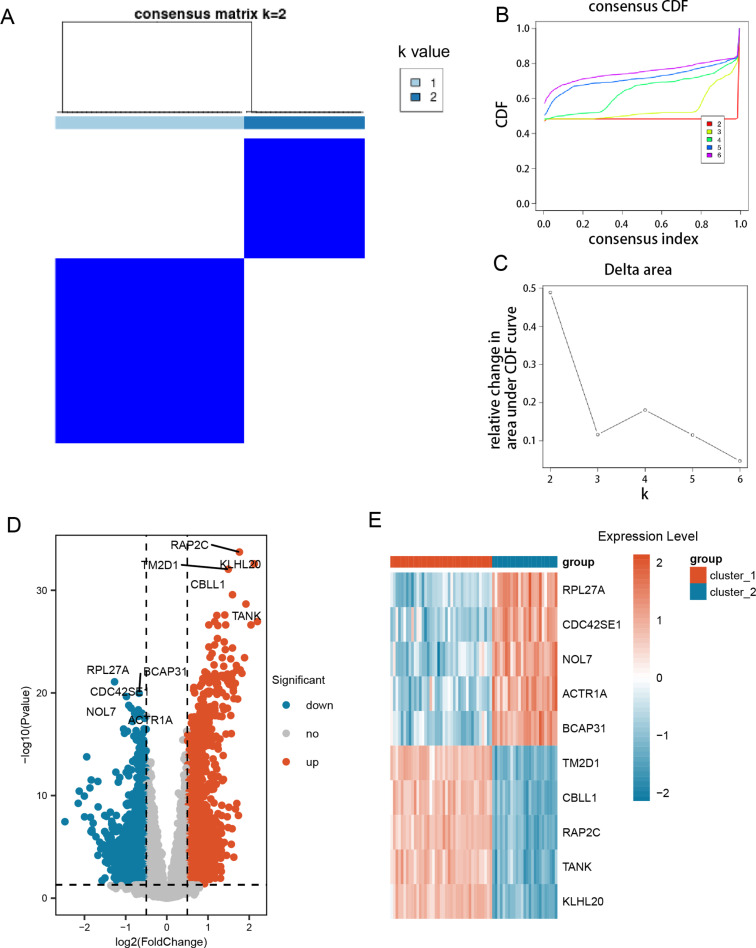
SLE sample clustering based on GRGs and differential analysis between clusters. (A) Consistency clustering heatmap for k = 2. The values of the consistency matrix are represented from 0 (unlikely to cluster together) to 1 (always clustered together), with colors ranging from white to dark blue. (B) CDF cumulative distribution curve of consistency clustering. (C) Delta Area graphs for k values from 2 to 6. (D) Volcano plot showing DEGs between cluster_1 and cluster_2 groups. (E) Heatmap showing the expression of top-ranked DEGs across samples of different subclusters.

We clustered the SLE patient samples using GRGs (S2 Table). We analyzed the consensus clustering heatmap (Fig 2A), the consensus CDF curve (Fig 2B), and the delta area curve (Fig 2C). This analysis indicated that the optimal number of clusters is 2, resulting in the division of SLE patient samples into two subclusters: cluster_1 and cluster_2.

We identified a total of 2,681 DEGs through the comparison of the subclusters. These DEGs exhibited statistically significant differences between the two groups (adjusted p < 0.05). In cluster_1, 1,458 genes were upregulated, while 1,223 genes were downregulated (S3 Table). All DEGs were visualized using a volcano plot (Fig 2D). Furthermore, a heatmap illustrated the expression levels of the top-ranked genes across the different subclusters, including TM2D1, CBLL1, RAP2C, TANK, KLHL20, RPL27A, CDC42SE1, NOL7, ACTR1A, and BCAP31 (Fig 2E).

### GO and KEGG pathway enrichment analysis

**Fig 3:**
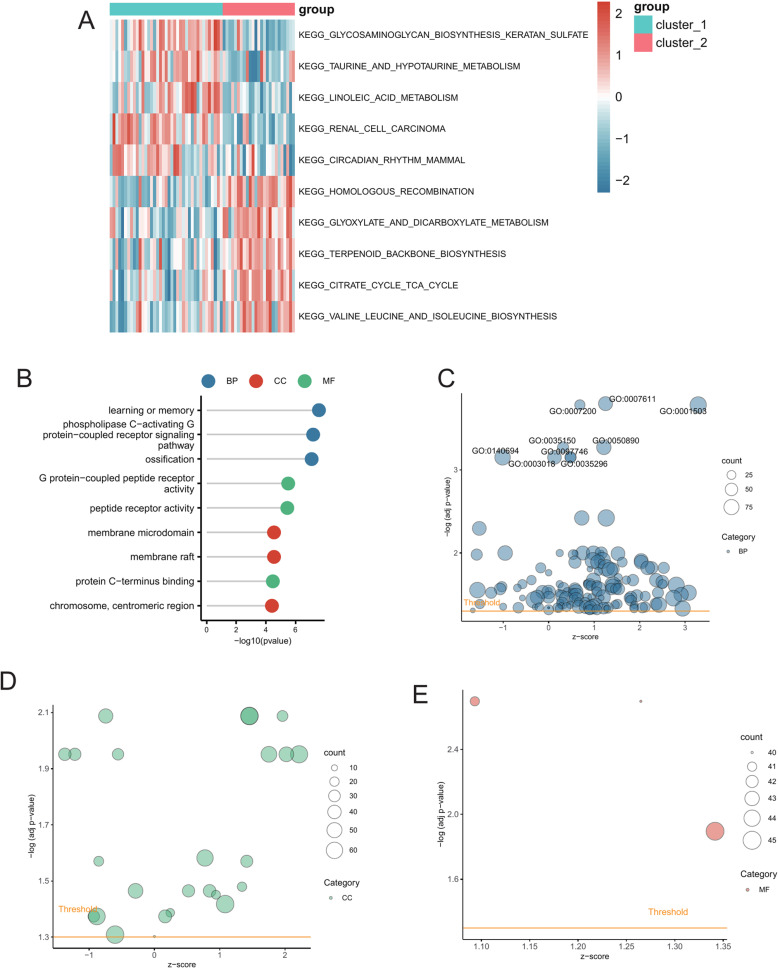
GSVA enrichment analysis between cluster_1 and cluster_2 and enrichment analysis of differential genes between subclusters. (A) Heatmap showing the enrichment of differential pathways between the two subclusters. (B) GO enrichment analysis of DEGs between subclusters. (C) Bubble plot showing the BP enrichment pathways of DEGs between subclusters. (D) Bubble plot showing the CC enrichment pathways of DEGs between subclusters. (E) Bubble plot showing the MF enrichment pathways of DEGs between subclusters.

We conducted GSVA analysis to explore the functional annotations of SLE and assess the differences in pathway expression between two clusters. The GSVA analysis identified several differentially expressed pathways. These pathways were visualized using heatmaps. The cluster_1 group exhibited significantly lower expression levels of KEGG RENAL CELL CARCINOMA and KEGG LINOLEIC ACID METABOLISM compared to the control group, whereas the KEGG CITRATE CYCLE TCA CYCLE and KEGG HOMOLOGOUS RECOMBINATION pathways exhibited significantly higher expression levels (Fig 3A, S4 Table).

We performed enrichment analysis of GO terms to investigate the biological functions associated with GRGs differences (S5 Table). The GO results indicated enrichment in several BP such as learning or memory (GO:0007611), phospholipase C-activating G protein-coupled receptor signaling pathway (GO:0007200), and ossification (GO:0001503). Additionally, we observed enrichment in CC like membrane raft (GO:0045121), membrane microdomain (GO:0098857), and chromosome, centromeric region (GO:0000775). Furthermore, MF included G protein-coupled peptide receptor activity (GO:0008528), peptide receptor activity (GO:0001653), and protein C-terminus binding (GO:0008022) (Fig 3B–3E).

### Immune infiltration analysis

**Fig 4:**
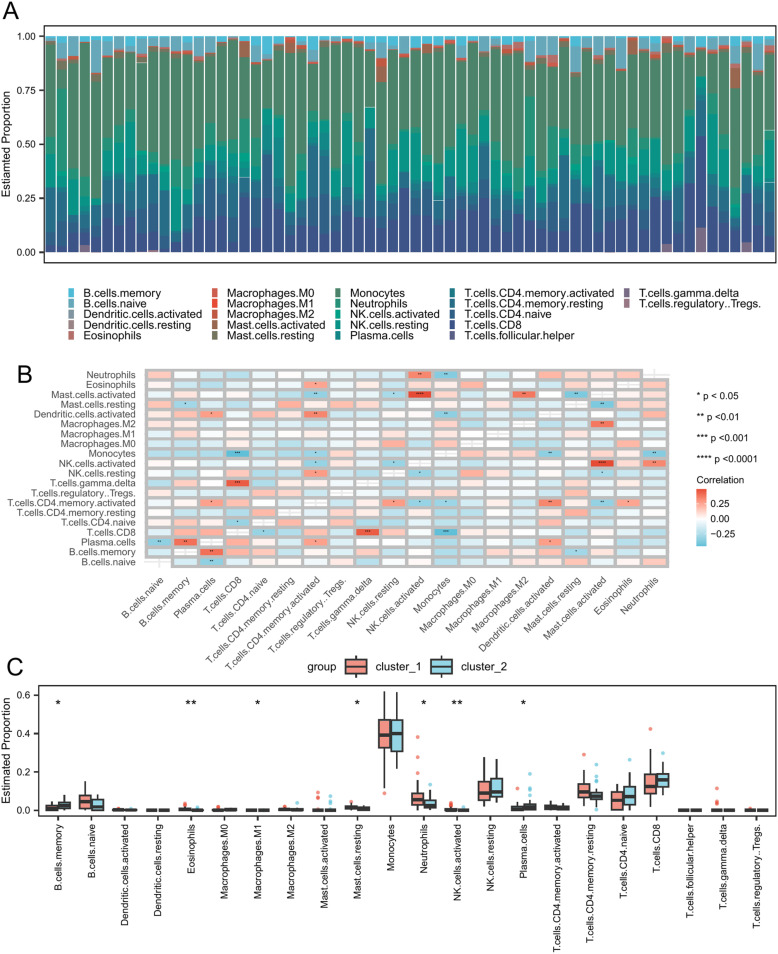
Immune infiltration analysis between cluster_1 and cluster_2. (A) Stacked bar chart of immune cells in SLE. (B) Heatmap displayed the correlation between immune cells in SLE. (C) Difference in estimated immune cell infiltration proportions between cluster_1 and cluster_2. Asterisks indicate p-values: ****p <  0.0001, ***p <  0.001, **p <  0.01, * p <  0.05.

We first used CIBERSORTx to analyze immune cell infiltration proportions in all samples. Our analysis revealed that monocyte had the highest proportion across all samples (Fig 4A). We also examined the correlations among immune cells and found that most exhibited negative relationships (Fig 4B).

To investigate the association between cluster_1 and cluster_2 and infiltrating immune cells, we conducted immune infiltration box plots for the two groups. Of the 28 immune cell types, 7 displayed significantly different infiltration levels between the two groups (p < 0.05) (S6 Table). Specifically, three immune cell types—resting mast cells, eosinophils, and neutrophils—had significantly higher infiltration levels in cluster_1 than in cluster_2 (Fig 4C).

### SLE-related DEGs and GSEA analysis

**Fig 5:**
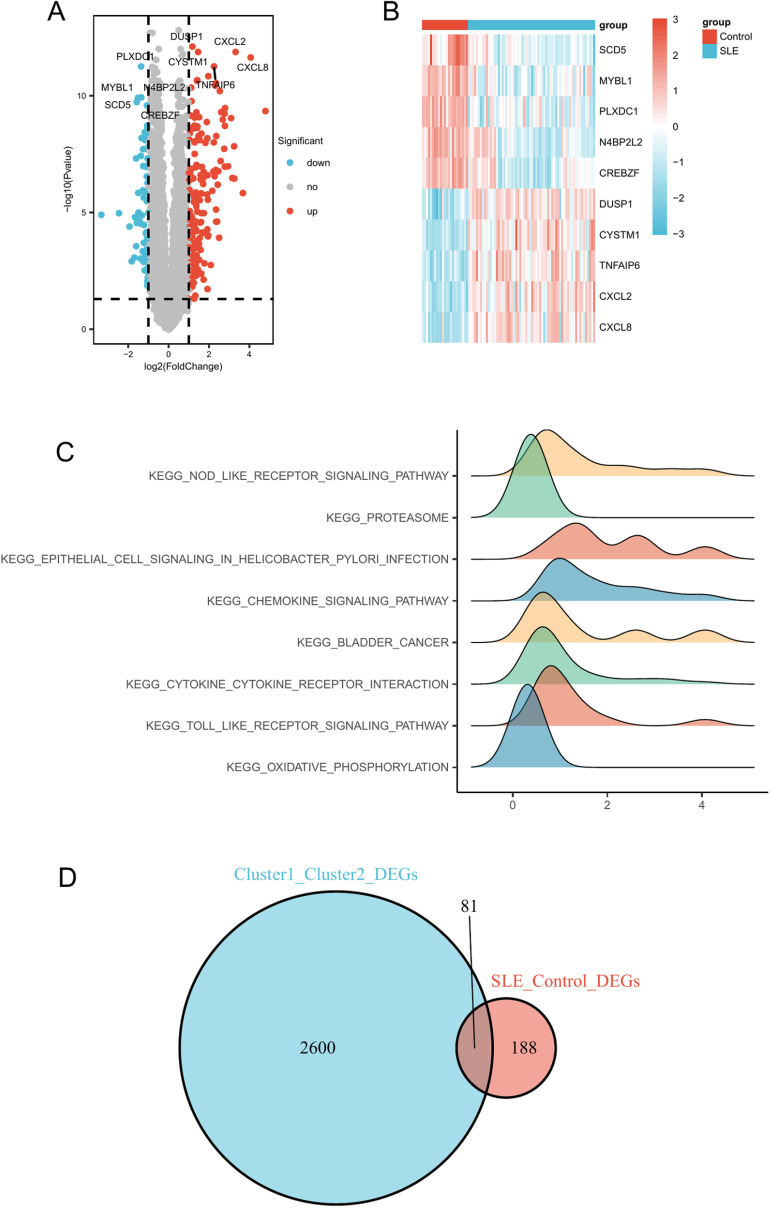
Expression of SLE-related DEGs. (A) Volcano plot showing DEGs between SLE and Control samples. (B) Heatmap showing the most significant DEGs between SLE and Control samples. (C) Ridge plot of GSEA analysis for DEGs. (D) Venn diagram showing the overlap of DEGs between SLE and Control groups, and DEGs between subclusters.

We identified a total of 269 DEGs by comparing SLE samples with a control group. These genes exhibited statistically significant differences between the two groups (adjusted p < 0.01). In SLE samples, 187 genes were upregulated and 82 genes were downregulated (S7 Table). All DEGs were visualized using a volcano plot (Fig 5A). Furthermore, a heatmap illustrated the expression levels of the top-ranked genes (DUSP1, CYSTM1, TNFAIP6, CXCL2, CXCL8, SCD5, MYBL1, PLXDC1, N4BP2L2, CREBZF) across the samples (Fig 5B).

To investigate the potential mechanisms underlying the DEGs, we performed a GSEA. Using the MSigDB, we selected the most significantly enriched signaling pathways based on their Normalized Enrichment Score (NES) (S8 Table). The GSEA significantly enriched pathways included the NOD-like receptor signaling pathway, proteasome, epithelial cell signaling in Helicobacter pylori infection, cytokine-cytokine receptor interaction, Toll-like receptor signaling pathway, and oxidative phosphorylation (Fig 5C).

By intersecting the DEGs from different clusters with those related to SLE, we identified 81 glucocorticoid-related DEGs, which we considered to be hub genes (Fig 5D, S9 Table).

### Identification of hub genes

**Fig 6:**
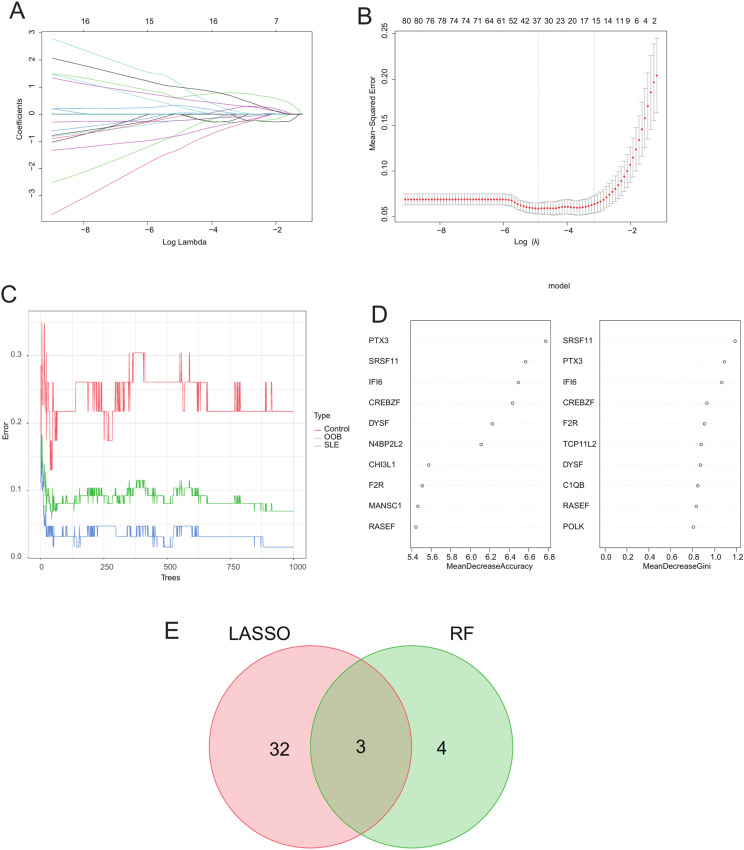
Results of machine learning for hub gene selection. (A) The trajectory of the independent variables in LASSO regression. (B) The confidence intervals for each lambda in LASSO regression. (C) Comparison of RF error rates with the number of classification trees. (D) The top 10 hub genes in the RF algorithm ranked by two types of importance. (E) Venn diagram illustrated the intersection of hub genes identified by LASSO and RF selection. RF, random forest; LASSO, least absolute shrinkage and selection operator.

Using LASSO regression analysis, we identified 35 hub genes (Fig 6A and 6B). Using the RF algorithm and the feature weights MDA and MDG, we selected 7 hub genes (Fig 6C and 6D). Finally, we found that the hub genes identified by each method intersected. This led to the identification of 3 hub genes: PTX3, DYSF and F2R (Fig 6E).

### GO and KEGG pathway enrichment analysis

**Fig 7:**
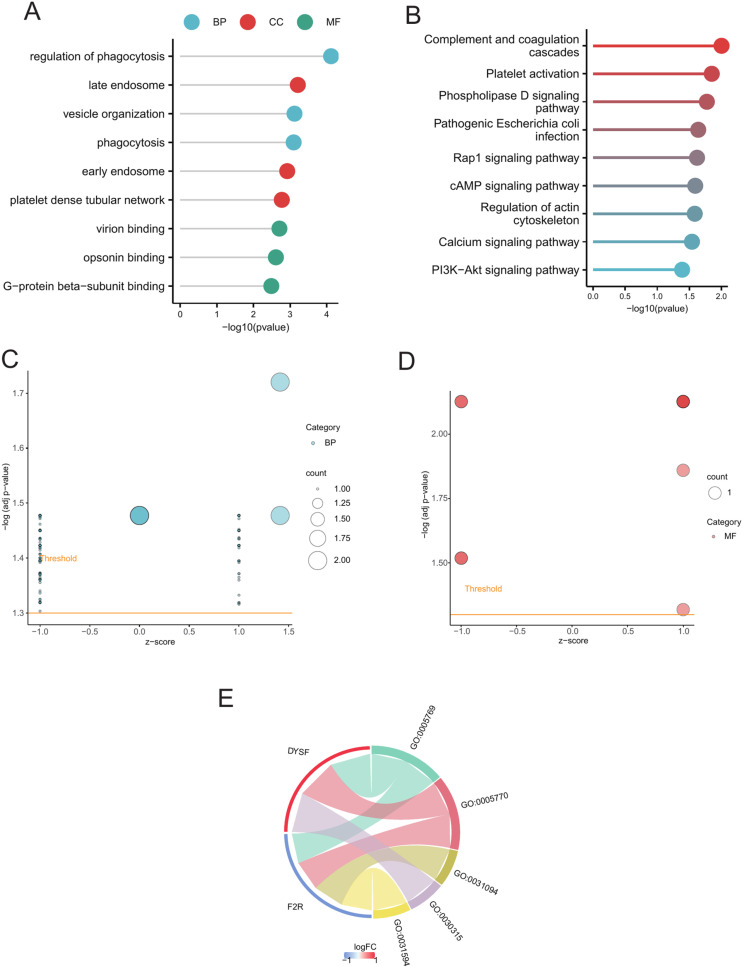
Enrichment analysis based on hub genes. (A) GO enrichment analysis. (B) KEGG enrichment analysis (C) Bubble plot showing BP pathway enrichment analysis. (D) Bubble plot showing MF pathway enrichment analysis. (E) Chord diagram showing BP pathway enrichment analysis.

We conducted enrichment analyses of GO terms (S10 Table) and KEGG pathways (S11 Table) to investigate the biological functions linked to differential expression associated with glucocorticoids. The GO analysis revealed that these genes were enriched in several biological processes, including the regulation of phagocytosis (GO:0050764), vesicle organization (GO:0016050), and late and early endosome formation (GO:0005770 and GO:0005769). Additionally, they were involved in the platelet dense tubular network (GO:0031094), virion binding (GO:0046790), opsonin binding (GO:0001846), and G-protein beta-subunit binding (GO:0031681) (Fig 7A, 7C and 7E). The enriched KEGG pathways included complement and coagulation cascades (hsa04610), platelet activation (hsa04611), and the phospholipase D signaling pathway (hsa04072) (Fig 7B).

#### Validation of hub genes expression and construction and validation of nomograms.

**Fig 8:**
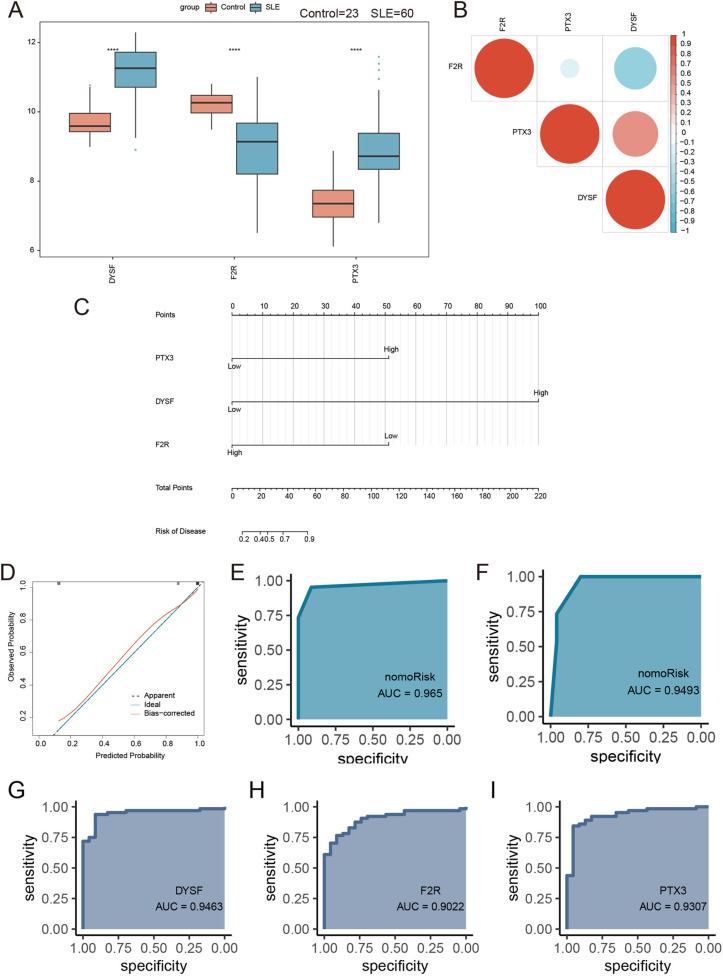
Expression patterns of hub genes in SLE-Control groups and construction and validation of a nomogram based on hub genes. (A) Hub genes expression boxplot. (B) Hub genes correlation heatmap. (C) Nomogram. (D) Nomogram standard curve. (E) ROC curve of the nomogram. (F) ROC curve of the nomogram validated with external dataset. ROC curve of hub gene DYSF (G), F2R (H) and PTX3(I).

We conducted a comparison of hub genes expression between the two groups and found significant differences. Specifically, the hub genes PTX3 and DYSF were significantly upregulated in SLE compared to the control group, whereas F2R was significantly downregulated (Fig 8A). To examine the relationships between the hub genes, we created a heatmap, which showed that F2R was negatively correlated with both PTX3 and DYSF (Fig 8B).

We developed a nomogram model for diagnosing SLE using the feature genes PTX3, DYSF and F2R (Fig 8C). We then assessed its predictive ability using a calibration curve. The calibration curve indicated minimal discrepancies between the actual and predicted SLE risk, demonstrating the model’s high accuracy (Fig 8D). ROC curve analysis also confirmed the model’s accuracy (ROC >  0.9) (Fig 8E). Furthermore, ROC curve analysis using the external dataset GSE81622 corroborated the model’s accuracy (ROC >  0.9) (Fig 8F). The ROC curves of the hub genes DYSF (Fig 8G), F2R (Fig 8H), and PTX3 (Fig 8I) were all greater than 0.9.

### Immune infiltration analysis

**Fig 9:**
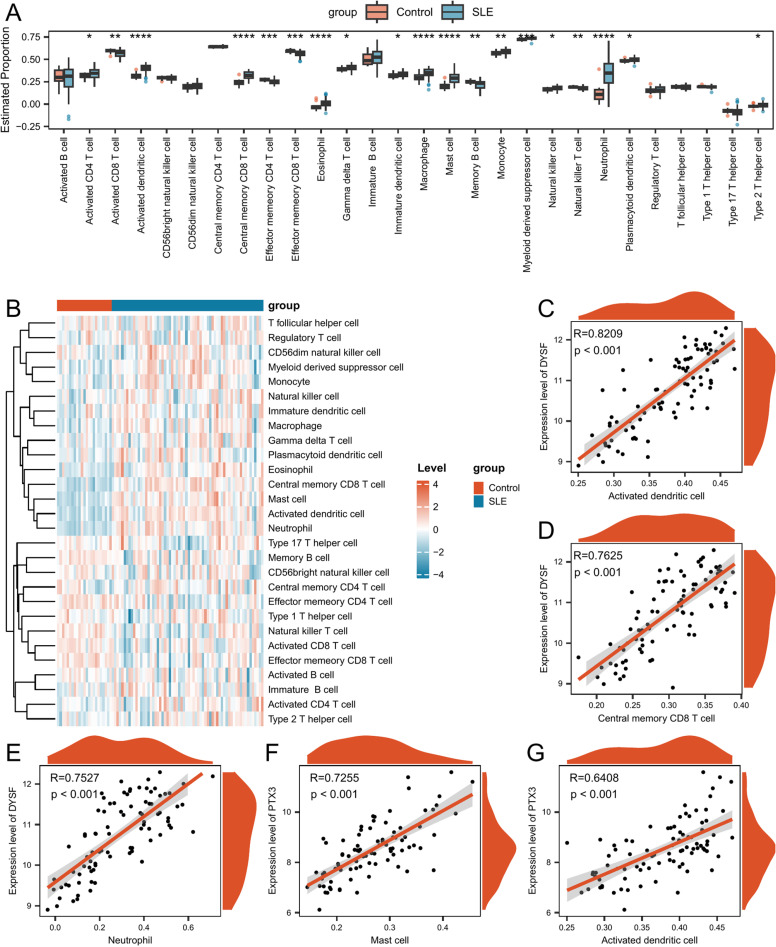
Immune infiltration analysis between SLE and control groups. (A) Difference in estimated immune cell infiltration proportions. (B) Heatmap displaying the changes in immune infiltration levels. (C) Scatter plot of PTX3 vs. Activated CD8 T cells. (D) Scatter plot of DYSF vs. Activated CD8 T cells. (E) Scatter plot of PTX3 vs. Central memory CD8 T cells. (F) Scatter plot of DYSF vs. Central memory CD8 T cells. (G) Scatter plot of F2R vs. Central memory CD8 T cells. Asterisks indicate p-values: ****p <  0.0001, ***p <  0.001, **p <  0.01, * p <  0.05.

We investigated the association between SLE and control samples regarding infiltrating immune cells. Among the 28 types of immune cells, 19 exhibited significant differences in infiltration levels between the SLE and control groups (p < 0.05) (Fig 9A, S12 Table). Fourteen immune cells, including Central Memory CD8 T Cells, Activated CD4 T Cells, Gamma Delta T Cells, and Natural Killer Cells, exhibited significantly higher infiltration levels in the SLE group than in the control group (Fig 9A).

Fig 9B illustrated the difference in overall immune cell infiltration between the SLE and control groups. We also assessed the significant correlations between each hub gene and its corresponding immune cells. PTX3 was significantly correlated with Activated CD8 T Cells (R =  -0.445, p <  0.001) (Fig 9C); DYSF was significantly correlated with Activated CD8 T Cells (R =  -0.497, p <  0.001) (Fig 9D). PTX3 was significantly correlated with Central Memory CD8 T Cells (R =  0.552, p <  0.001) (Fig 9E). DYSF was significantly correlated with Central Memory CD8 T Cells (R =  0.762, p <  0.001) (Fig 9F). F2R was significantly correlated with Central Memory CD8 T Cells (R =  -0.471, p <  0.001) (Fig 9G).

### Signaling pathways related to hub genes

**Fig 10:**
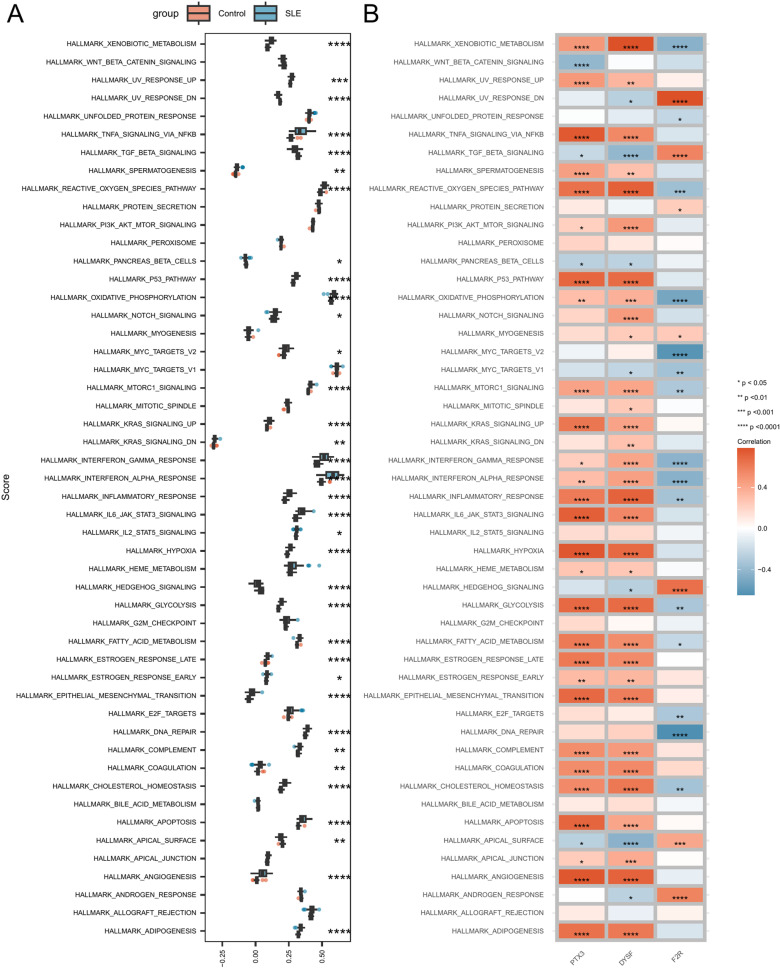
Correlation between hub genes and the 50 Hallmark signaling pathways. (A) Comparison of the 50 Hallmark signaling pathways between the SLE group and the control group. (B) Correlation between hub genes and the 50 Hallmark signaling pathways. Asterisks indicate p-values: ****p <  0.0001, ***p <  0.001, **p <  0.01, * p <  0.05.

We further investigated the differences between SLE patients and the control group using GSVA for 50 Hallmark signaling pathways. In SLE patients, 30 Hallmark pathways were significantly upregulated, including HALLMARKADIPOGENESIS, HALLMARKANGIOGENESIS, HALLMARKAPOPTOSIS, HALLMARKCHOLESTEROLHOMEOSTASIS, HALLMARKCOAGULATION, HALLMARKCOMPLEMENT, HALLMARKDNAREPAIR, HALLMARKEPITHELIALMESENCHYMALTRANSITION, HALLMARKESTROGENRESPONSEEARLY, HALLMARKESTROGENRESPONSELATE, HALLMARKFATTYACIDMETABOLISM, HALLMARKGLYCOLYSIS, HALLMARKHYPOXIA, HALLMARKIL2STAT5SIGNALING, HALLMARKIL6JAKSTAT3SIGNALING, HALLMARKINFLAMMATORYRESPONSE, HALLMARKINTERFERONALPHARESPONSE, HALLMARKINTERFERONGAMMARESPONSE, HALLMARKKRASSIGNALINGDN, HALLMARKKRASSIGNALINGUP, HALLMARKMTORC1SIGNALING, HALLMARKMYCTARGETSV2, HALLMARKNOTCHSIGNALING, HALLMARKOXIDATIVEPHOSPHORYLATION, HALLMARKP53PATHWAY, HALLMARKREACTIVEOXYGENSPECIESPATHWAY, HALLMARKSPERMATOGENESIS, HALLMARKTNFASIGNALINGVIANFKB, HALLMARKUVRESPONSEUP, and HALLMARKXENOBIOTICMETABOLISM. Five pathways were significantly downregulated, including HALLMARKAPICALSURFACE, HALLMARKHEDGEHOGSIGNALING, HALLMARKPANCREASBETACELLS, HALLMARKTGFBETASIGNALING, and HALLMARKUVRESPONSEDN (Fig 10A, S13 Table).

We also analyzed the correlation between hub genes and the 50 Hallmark signaling pathways. PTX3 was associated with several pathways, including HALLMARKADIPOGENESIS and HALLMARKALLOGRAFTREJECTION. DYSF was also associated with several pathways, including HALLMARKADIPOGENESIS and HALLMARKALLOGRAFTREJECTION (Fig 10B).

### Network construction of hub mRNAs

**Fig 11:**
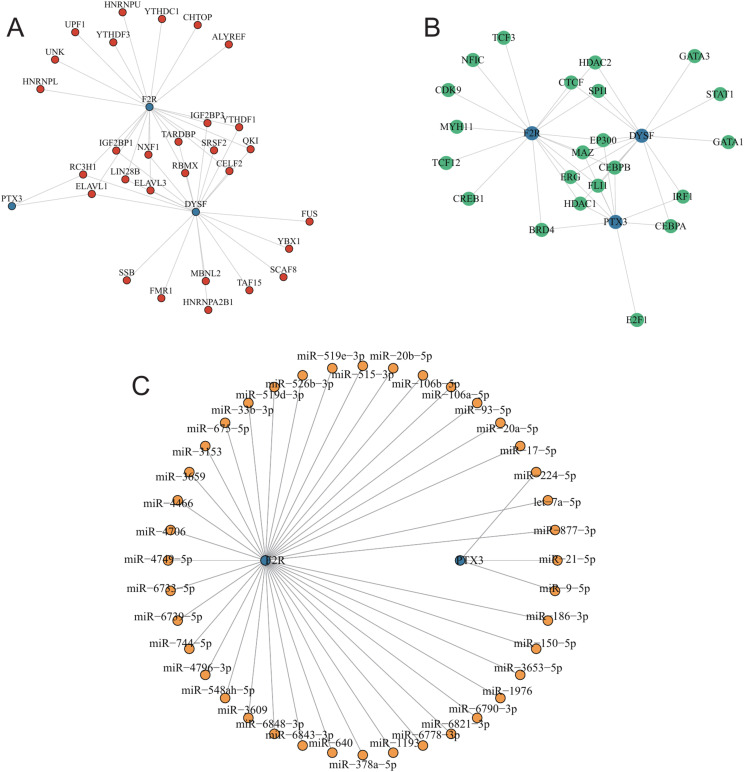
Construction of the regulatory network based on hub genes. (A) RBP-mRNA regulatory network. (B) mRNA-TF network. (C) miRNA-mRNA network.

We utilized the StarBase online database to search for three hub mRNAs and download their corresponding mRNA/RBP pairs, which are formed by the binding of RBPs with mRNA. Using the relationships from the online dataset, we constructed an RBP-mRNA network. This network consisted of 32 nodes, including 29 RBPs and 3 mRNAs, connected by 44 edges. Detailed information about the nodes and interactions can be found in S14 Table, while the network was illustrated in Fig 11A.

Next, to elucidate the potential molecular mechanisms of hub genes in SLE, we constructed an mRNA–transcription factor (TF) interaction network. The Cytoscape-generated network diagram included 3 mRNAs and 22 TFs (Fig 11B).

We then constructed an mRNA–miRNA interaction network. The Cytoscape-generated network diagram included 2 mRNAs and 40 miRNAs, totaling 42 nodes and 40 edges (Fig 11C).

### Drug sensitivity analysis and molecular docking

**Fig 12:**
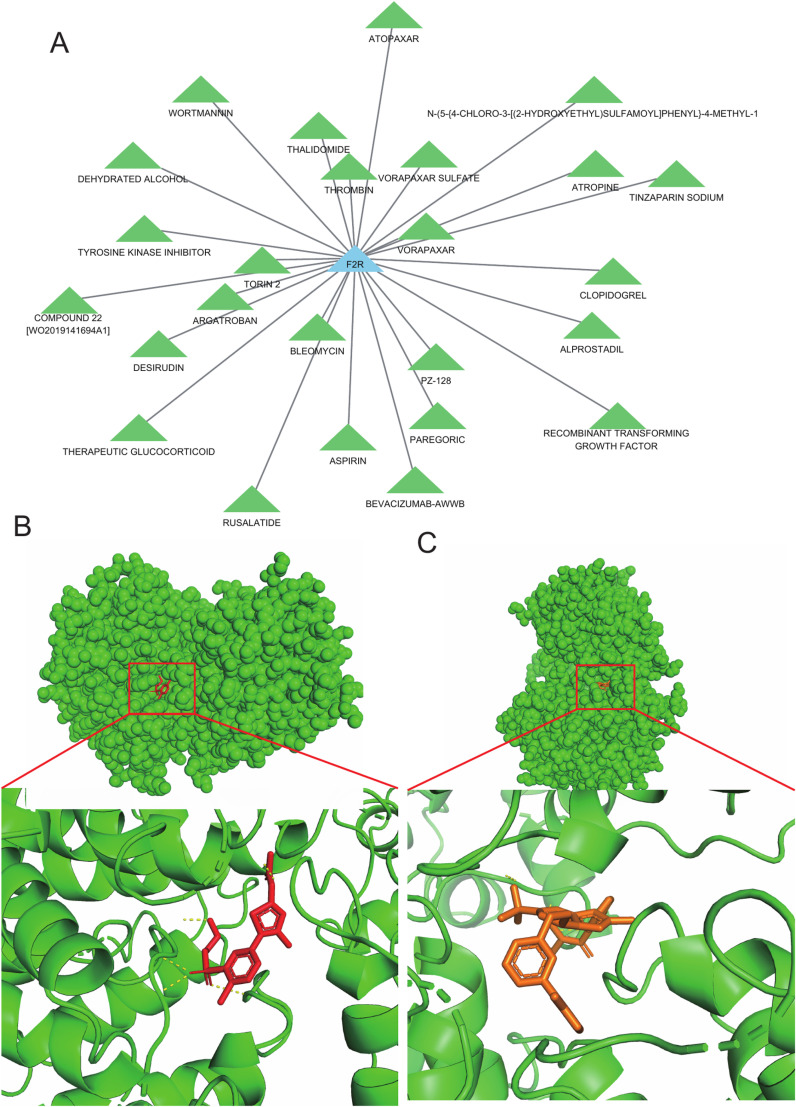
Analysis of drug sensitivity and molecular docking. (A)Drug sensitivity analysis of F2R. (B) Molecular docking of F2R with N-(5-{4-CHLORO-3-[(2-HYDROXYETHYL) SULFAMOYL] PHENYL}-4-METHYL-1,3-THIAZOL-2-YL) ACETAMIDE. (C) Molecular docking of F2R with COMPOUND 22 [WO2019141694A1].

We conducted a drug sensitivity analysis on three hub genes identified in DGIdb. Our findings revealed that only the hub gene F2R had a significant correlation with drugs, while the other two hub genes showed no drug-related correlations. As illustrated in the drug-gene interaction network, 25 drugs had varying degrees of regulatory effects on the hub genes (Fig 12A, S15 Table).

For the hub gene F2R, we conducted molecular docking with drugs predicted by DGIdb that have high interaction scores and known 3D structures. We discovered that the binding energies of F2R with two drugs, COMPOUND 22 [WO2019141694A1] and N-(5-{4-CHLORO-3-[(2-HYDROXYETHYL) SULFAMOYL] PHENYL}-4-METHYL-1,3-THIAZOL-2-YL) ACETAMIDE, were both less than zero (Table 1). This finding showed that these drugs bind strongly to the hub gene F2R, suggesting that this interaction may lead to therapeutic effects by targeting the F2R protein (Fig 12B and 12C).

**Table 1:** Basic information on molecular docking of drugs with target Proteins.

Molecular name	Targets	PDB ID/Protein Accession	Binding energy(kcal/Mol)
N-(5-{4-CHLORO-3-[(2-HYDROXYETHYL) SULFAMOYL] PHENYL}-4-METHYL-1,3-THIAZOL-2-YL) ACETAMIDE.	F2R	6HH4	-6.045
COMPOUND 22 [WO2019141694A1]	F2R	6HH4	-7.232

## Discussion

SLE is a complex autoimmune disease, and its causes are not yet fully understood. Current research focuses on the expression of GRGs in SLE. However, the specific mechanisms and regulatory roles of GRGs in SLE had not been thoroughly explored. Therefore, we used bioinformatics analysis and machine learning to explore the molecular characteristics of GRGs in SLE.

Through consensus clustering analysis, we identified two distinct clusters based on GRG expression, which illustrated different regulatory patterns in SLE patients. The findings showed that gene expression levels in SLE patients are elevated compared to normal individuals, highlighting the crucial role of GRGs in the development of SLE. This stratification offers a promising avenue for personalized treatment, as different subclusters may exhibit unique clinical presentations and responses to therapy [36]. Investigating the clinical significance of these subclusters could enhance our understanding of disease heterogeneity in SLE [37]. By analyzing the molecular mechanisms underpinning the differences observed between subclusters, researchers may identify specific therapeutic targets that are more relevant for each group [38].

Our gene expression analysis revealed 269 DEGs when comparing SLE patients to the control group, with 187 genes being upregulated and 82 downregulated. These findings showed a distinct gene expression profile linked to SLE. The characterization of these DEGs offered a foundation for understanding how specific genes may contribute to the inflammatory processes and autoimmunity observed in SLE [39].

GSEA showed significant enrichment of pathways such as the NOD-like receptor signaling pathway and proteasome pathway in SLE patients. The activation of these pathways suggests a heightened immune response and potential alterations in cellular homeostasis. Specifically, the NOD-like receptor signaling pathway is known to play a crucial role in detecting intracellular pathogens and modulating inflammatory responses, which may contribute to the sustained inflammation characteristic of SLE [40]. The proteasome pathway plays an important role in protein degradation and immune response, especially in autoimmune diseases like SLE, where abnormalities in protein degradation may lead to the overactivation of immune cells [41]. The cytokine-receptor interaction pathway is crucial in SLE, as immune system abnormalities are commonly observed in SLE patients, characterized by excessive cytokine secretion. Cytokines play a key role in the pathophysiology of SLE and may trigger or exacerbate immune system dysregulation [42]. The Toll-like receptor (TLR) signaling pathway is one of the key pathways in SLE, with TLR receptors playing an important role in recognizing both self and foreign antigens. Aberrant activation of TLRs is closely associated with the onset and progression of SLE, particularly in the context of immune system dysregulation [43].

The analysis using CIBERSORTx revealed a significant increase in monocyte infiltration in SLE patients, with 19 immune cell types demonstrating heightened presence compared to controls. This finding highlighted the critical role of immune cell dynamics in the pathogenesis of SLE, suggesting that immune cell infiltration could be a key driver of disease progression [44]. The increased monocyte levels may indicate an ongoing inflammatory response, potentially exacerbating tissue damage and contributing to the clinical manifestations seen in SLE [13]. This finding aligns with existing literature that emphasizes the role of immune cell dynamics in SLE pathogenesis [45]. We analyzed immune cell infiltration in SLE samples and found that monocyte infiltration was the highest among all samples. These results suggested that GRGs were key factors in regulating the onset of SLE and the immune infiltration status.

Our research identified three hub genes (PTX3, DYSF, F2R) associated with SLE. PTX3 plays a key role in innate immune responses, inflammation, and tissue injury and remodeling [46]. Increasing evidence shows that PTX3 is involved in the occurrence and development of multiple autoimmune diseases, such as rheumatoid arthritis and SLE [47,48]. In autoimmune diseases such as SLE, PTX3 may promote the clearance of immune complexes or regulate immune responses by activating or modulating the complement pathway [49]. DYSF is involved in membrane repair and inflammation regulation in endothelial cells and leukocytes [50]. DYSF dysfunction may lead to impaired phagocytic activity of immune cells such as macrophages, particularly affecting cell membrane repair and the endocytic process, which could impact the immune response in SLE patients [51]. F2R also plays an important role in coagulation and inflammatory responses during injury [52]. Activation of F2R may promote the initiation of the coagulation cascade, affecting coagulation function in SLE patients, particularly playing a role in immune complex-induced vascular injury [53]. Based on these hub genes, we developed a diagnostic nomogram model which is capable of accurately predicting outcomes related to SLE. This model performed well in calibration, ROC analysis, and external validation. Our results provide important references for future research and clinical practice.

In our drug sensitivity analysis, we found a significant correlation between the hub gene F2R and 25 distinct drugs or molecular compounds. Our findings indicated that both COMPOUND 22 [WO2019141694A1] and N-(5-{4-CHLORO-3-[(2-HYDROXYETHYL) SULFAMOYL] PHENYL}-4-METHYL-1,3-THIAZOL-2-YL) ACETAMIDE have a strong binding affinity for F2R, which may lead to potential therapeutic effects through the targeted interaction with the F2R protein. COMPOUND 22 [WO2019141694A1], also known as PI4KIIIbeta-IN-11, is an inhibitor of PI4KIIIβ. In the context of SLE treatment, the potential of PI4KIIIβ inhibitors lies in their ability to modulate immune responses by affecting membrane trafficking and signal transduction [54]. N-(5-{4-CHLORO-3-[(2-HYDROXYETHYL) SULFAMOYL] PHENYL}-4-METHYL-1,3-THIAZOL-2-YL) ACETAMIDE, also known as PIK-93, is a selective inhibitor of phosphoinositide 3-kinase (PI3K), primarily exerting its effects by inhibiting the PI3K-Akt signaling pathway [55]. Notably, the PI3K-Akt pathway is implicated in the enrichment results of three key genes in our study, suggesting that PIK-93 may play a role in the treatment of SLE by targeting F2R and modulating the PI3K-Akt pathway. This relationship points to the potential of F2R as a biomarker for predicting therapeutic responses in SLE patients. This approach aligns with the growing emphasis on personalized medicine in autoimmune disorders, where understanding genetic predispositions and molecular signatures can significantly influence treatment outcomes [56].

This study has several limitations. Firstly, the relatively small sample size may restrict the generalizability of our findings, as a larger cohort could provide more robust insights into the genetic and immunological underpinnings of SLE. Additionally, the absence of experimental validation limits the capacity to confirm the functional relevance of the identified DEGs and pathways. Furthermore, the lack of comprehensive clinical data restricts our ability to correlate the identified biomarkers with disease severity and patient outcomes. Future investigations should aim to address these limitations by incorporating larger and more diverse populations, alongside experimental validation to substantiate the observed associations.

## Conclusion

We found that the hub genes PTX3, DYSF and F2R were closely associated with SLE. The research emphasized the importance of immune cell infiltration and signaling pathways in the pathogenesis of SLE. Our study offered new insights into the molecular basis of SLE and identified potential therapeutic targets for its diagnosis and treatment.

## Supporting information

S1 Table Glucocorticoid related genes. (CSV)

S2 Table Consensus group. (CSV)

S3 Table Differential gene expression analysis results. (CSV)

S4 Table GSVA pathway. (CSV)

S5 Table Enrichment analysis of GO. (CSV)

S6 Table CIBERSORTx results. (CSV)

S7 Table Differential gene expression analysis results. (CSV)

S8 Table GSEA result. (CSV)

S9 Table Lasso gene RF hub gene. (CSV)

S10 Table Enrichment analysis of GO. (CSV)

S11 Table Enrichment analysis of KEGG. (CSV)

S12 Table Statistically significant immune cell differences. (CSV)

S13 Table Hallmark significant GSVA results. (CSV)

S14 Table RBP table. (CSV)

S15 Table DGIdb. (CSV)
